# Clinical Outcomes of low-voltage area-guided left atrial linear ablation for non-paroxysmal atrial fibrillation patients

**DOI:** 10.1371/journal.pone.0260834

**Published:** 2021-12-02

**Authors:** Hao-Tien Liu, Chia-Hung Yang, Hui-Ling Lee, Po-Cheng Chang, Hung-Ta Wo, Ming-Shien Wen, Chun-Chieh Wang, San-Jou Yeh, Chung-Chuan Chou

**Affiliations:** 1 Division of Cardiology, Department of Internal Medicine, Chang Gung Memorial Hospital, Linkou Branch, Taoyuan, Taiwan; 2 Department of Anesthesia, Chang Gung Memorial Hospital, Taipei Branch, Taipei, Taiwan; 3 Chang Gung University College of Medicine, Taoyuan, Taiwan; University of Minnesota, UNITED STATES

## Abstract

**Background:**

The therapeutic effect of low-voltage area (LVA)-guided left atrial (LA) linear ablation for non-paroxysmal atrial fibrillation (non-PAF) is uncertain. We aimed to investigate the efficacy of LA linear ablation based on the preexisting LVA and its effects on LA reverse remodeling in non-PAF patients.

**Methods:**

We retrospectively evaluated 145 consecutive patients who underwent radiofrequency catheter ablation for drug-refractory non-PAF. CARTO-guided bipolar voltage mapping was performed in atrial fibrillation (AF). LVA was defined as sites with voltage ≤ 0.5 mV. If circumferential pulmonary vein isolation couldn’t convert AF into sinus rhythm, additional LA linear ablation was performed preferentially at sites within LVA.

**Results:**

After a mean follow-up duration of 48 ± 33 months, 29 of 145 patients had drugs-refractory AF/LA tachycardia recurrence. Low LA emptying fraction, large LA size and high extent of LVA were associated with AF recurrence. There were 136 patients undergoing LA linear ablation. The rate of linear block at the mitral isthmus was significantly higher via LVA-guided than non-LVA-guided linear ablation. Patients undergoing LVA-guided linear ablation had larger LA size and higher extent of LVA, but the long-term AF/LA tachycardia-free survival rate was higher than the non-LVA-guided group. The LA reverse remodeling effects by resuming sinus rhythm were noted even in patients with a diseased left atrium undergoing extensive LA linear ablation.

**Conclusions:**

LVA-guided linear ablation through targeting the arrhythmogenic LVA and reducing LA mass provides a better clinical outcome than non-LVA guided linear ablation, and outweighs the harmful effects of iatrogenic scaring in non-PAF patients.

## Introduction

Because circumferential pulmonary vein isolation (CPVI) alone for non-paroxysmal atrial fibrillation (non-PAF) is associated with a low successful rate, substrate modification has been incorporated into radiofrequency catheter ablation (RFCA) treatment to improve clinical outcomes [1, 2]. Linear ablation with partially compartmentalization of the atria may prevent formation of macroreentrant circuits and thus recurrence of atrial fibrillation (AF). However, the outcomes of additional linear ablation varies in the hands of different operators [3]. Meantime, incomplete linear ablation lesions without bidirectional block and localized scar-related reentrant left atrial tachycardia (LAT) from previous ablation or structural heart disease were the main causes of suboptimal outcomes in the STAR-AF II trial [4]. It implies that achieving linear lesion contiguity via transvenous RFCA remains challenging technically. Low-voltage area (LVA) reflects endocardial scar and atrial tissue with different degree of structural defect and remodeling. LVA can perpetuate AF by either facilitating reentry or acting as a boundary crucial for rotors anchoring, and the presence of LVA is an independent predictor of ablation failure for non-PAF [5] and PAF [6, 7]. For PAF patients, a recent published VOLCANO trial revealed that LVA presence strongly predicted AF recurrence, but LVA ablation had no beneficial impact on 1-year rhythm outcomes [8]. In contrast, for non-PAF ablation, substrate modification by targeting LVA has been proposed as an effective ablation approach, including patient-tailored [9], box-isolated [10], and homogenized [11] strategies. Efremidis et al. reported that targeted ablation of complex-fractionated electrograms within LVA after CPVI had a late AF recurrence rate of 28.6% [12]. Whether non-PAF patients benefit from additional LVA-guided linear ablation has not been reported yet. Eliminating AF induces left atrial (LA) reverse remodeling [13]. However, in patients with long-standing AF and a severely diseased left atrium, RFCA-induced injury could have deleterious effects on LA structure and function [14]. Whether an extensive linear ablation strategy applied to a diseased left atrium induces reverse remodeling or further deteriorates LA function in non-PAF patients remains unclear. In this study, we aimed to investigate the clinical outcomes of additional LVA-guided linear ablation in non-PAF patients and to analyze LA reverse remodeling after this procedure.

## Materials and methods

### Study population

We retrospectively evaluated 145 consecutive patients who underwent RFCA for drug-refractory non-PAF between July 2011 and July 2019 at our institution. In accordance with the HRS/EHRA/ECAS expert consensus statement, non-PAF was defined as continuous AF sustained for more than 7 days [15]. For all patients, detailed medical histories regarding AF and related cardiovascular and systemic conditions were obtained. On the basis of RFCA outcome, we divided patients into three groups: Group 1, no AF recurrence; Group 2, recurrence of AF/ LAT responsive to antiarrhythmic drugs (AADs); and Group 3, recurrence of AF/LAT refractory to AADs [16]. Patients were excluded if they had severe valvular disease requiring surgery, had received previous RFCA or prior surgical Cox maze procedure, or had LA appendage thrombosis. Transesophageal echocardiography and cardiac CT were performed in all patients before ablation to exclude the possibility of thrombi. All AADs were discontinued at least five half-lives before the study, with the exception of amiodarone, which was discontinued at least 3 months before the ablation procedure. The Institution Review Board of Chang Gung Memorial Hospital approved the study protocol (IRB No. 202001057B0), and written informed consent was obtained from all patients.

### Electrophysiological study and RFCA

All patients underwent RFCA under endotracheal intubation and general anesthesia. Heparin was administered to keep the activated clotting time > 300 s. RFCA was performed using a 3D electroanatomical mapping system (Carto 3, Biosense Webster, Diamond Bar, CA, USA) to support the creation and validation of ablation lesions. A 3.5-mm open-tip irrigated catheter (NaviStar Thermo-Cool, Biosense Webster) was percutaneously introduced through the right femoral vein for mapping and ablation. All electroanatomical mapping was performed in AF rhythm, and > 350 points were requested in each patient (mean 452 ± 220 points). We defined the area with a bipolar peak-to-peak voltage of < 0.5 mV as LVA [5]. LVA size was manually measured on each voltage map.

CPVI with confirmation of entrance block was verified in all patients. The ablation catheter was moved point by point in a dragging fashion to create successive lesions. If AF persisted or LAT occurred after CPVI, additional LA linear ablation was performed at the operator’s discretion. The roof, posterior mitral, anterior mitral, posterior (for box isolation), and anteroseptal lines were potential targets. We preferred performing linear ablation within LVA, especially at sites with complex-fractionated and/or high-frequency electrograms (the LVA-guided ablation group); whereas patients underwent at least one linear ablation not within the LVA were classified as the non-LVA guided ablation group. In the LVA-guided ablation group, the ablation lines were attempted to connect the CPVI lesions and/or the mitral annulus within the LVA at a distance as short as possible. Therefore, these linear ablation lesions could be within or at the border of LVA, depending on the distribution of LVA. External cardioversion was performed to restore sinus rhythm if RFCA failed to convert AF. The attempted endpoint of linear ablation was conduction block, validated by differential pacing with bidirectional reversal of the peri-mitral activation sequence and/or the recording of local separated double potentials at the entire ablation line [1, 17]. Non-pulmonary vein triggers that reinitiated AF were ablated as deemed necessary.

### Echocardiography

2D echocardiographic examinations were performed on the next day after RFCA, and serial echocardiographic examinations were performed at 1, 3, 6, and12 months and then every 6 months after RFCA. These examinations were performed using a commercially available ultrasound scanner (Vivid 9, General Electric Medical Health, Waukesha, WI, USA) with a 2.5-MHz phased-array transducer. The LA volume was measured using an apical 4-chamber view. The maximal LA volume (LAV_max_) was defined as the volume just before the mitral valve opening, and the minimal LA volume (LAV_min_) was defined as the smallest volume during ventricle diastole. The LA emptying fraction (LAEF) was calculated as (LAV_max_−LAV_min_)/LAV_max_ × 100% [18]. To evaluate the effects of AF ablation on LA structure and function, the differences of LA dimension (LAD), LAV_max_, LAV_min_, and LAEF between 1 day and 3 months after RFCA were calculated.

### Follow-up and definition of recurrence

Patients were followed up at 1 week, 1 month, 3 months, 6 months and every 3–6 months after RFCA and whenever required due to AF symptoms. Twelve-lead electrocardiograms and 24-h Holter ambulatory electrocardiograms were recorded after RFCA and when the patient experienced palpitation symptoms. Recurrence was defined as typical palpitation episodes for >30 seconds or atrial tachyarrhythmia on a 12-lead electrocardiogram, Holter monitoring, or pacemaker/implantable cardioverter-defibrillator interrogation records. Repeat RFCA was suggested to patients with AF/LAT recurrence after the 3-month blanking period.

### Statistical analysis

Continuous variables were summarized as mean ± standard deviation and categorical variables were represented using numbers and percentages. To analyze the effects of RFCA on echocardiographic measurements, we performed independent sample t test or analysis of variance procedures for continuous variables and the chi-square test or Fisher’s exact test for categorical variables. Survival curves of the freedom from AF/LAT recurrence were plotted via the Kaplan–Meier method, with the statistical significance between curves determined using the log-rank test. The two-tailed *P* value <0.05 was considered statistically significant.

## Results

### Patient characteristics and predictors of RFCA outcomes

Baseline clinical characteristics of the included patients are summarized in Table 1. The study population comprised 145 non-PAF patients (mean age 58 ± 13 years; mean duration of AF before RFCA 3.85 ± 3.53 years; and 72% male). The mean follow-up duration was 48 ± 33 months (median: 34 months). There were 63 (43.4%), 53 (36.6%) and 29 patients (20.0%) in Groups 1, 2, and 3, respectively. No significant difference was noted in age, gender, ablation times, and underlying medical diseases among groups except rheumatic heart disease. Group 3 had the longest AF duration (*P* = 0.014). AF conversion with RFCA procedures strongly predicted long-term AF-free outcome: In 65 (44.8%) patients with AF conversion by RFCA, only 3 (4.6%) patients were in Group 3; whereas in patients without AF conversion by RFCA, 28 of 80 (37.5%) patients were in Group 3 (*P* < 0.001).

**Table 1:** Baseline characteristics, clinical data, and ablation results of the study groups.

	All patients	Group 1	Group 2	Group 3	*p* value
Patient number	145	63 (43.4%)	53 (36.6%)	29 (20.0%)	.688
Follow/up (month)	48±33	48±34	50±34	44±29	
Age (years)	58±13	56±12	60±14	58±12	.278
Gender (male, %)	105 (72%)	49 (78%)	36 (68%)	20 (69%)	.446
BMI (kg/m^2^)	26.3±3.6	26.0±3.6	27.0±3.3	26.1±4.1	.290
AFD (years)	3.85±3.53	3.26±2.97	3.64±3.16	5.52±4.78	.014
CHA_2_DS_2_-VASc	1.73±1.42	1.42±1.39	2.00±1.34	1.87±1.52	.074
Hypertension (%)	79 (55%)	33 (52%)	34 (64%)	12 (41%)	.128
Diabetes mellitus (%)	27 (19%)	13 (21%)	12 (23%)	2 (7%)	.186
Dyslipidemia (%)	58 (40%)	22 (36%)	25 (48%)	11 (36%)	.332
CAD (%)	5 (3%)	0 (0%)	4 (8%)	1 (3%)	.067
Stroke (%)	19 (13%)	7 (11%)	6 (11%)	6 (21%)	.400
ESRD (%)	3 (2%)	1 (2%)	0 (0%)	2 (7%)	.149
RHD (%)	7 (5%)	0 (0%)	4 (8%)	3 (10%)	.019
SSS (%)	24 (17%)	7 (11%)	12 (23%)	5 (17%)	.249
COPD (%)	4 (3%)	1 (2%)	2 (4%)	1 (3%)	.534
Smoking (%)	12 (8%)	4 (6%)	4 (8%)	4 (14%)	.267
RFCA times	1.49±0.79	1.43±0.86	1.53±0.67	1.55±0.87	.715
AF conversion	65 (45%)	35 (56%)	27 (51%)	3 (10%)	< .001

AFD: atrial fibrillation duration; BMI: body-mass index; CAD: coronary artery disease; COPD: chronic obstructive pulmonary disease; ESRD: end-stage renal disease; RFCA: radiofrequency catheter ablation RHD: rheumatic heart disease; SSS: sick sinus syndrome.

**Fig 1:**
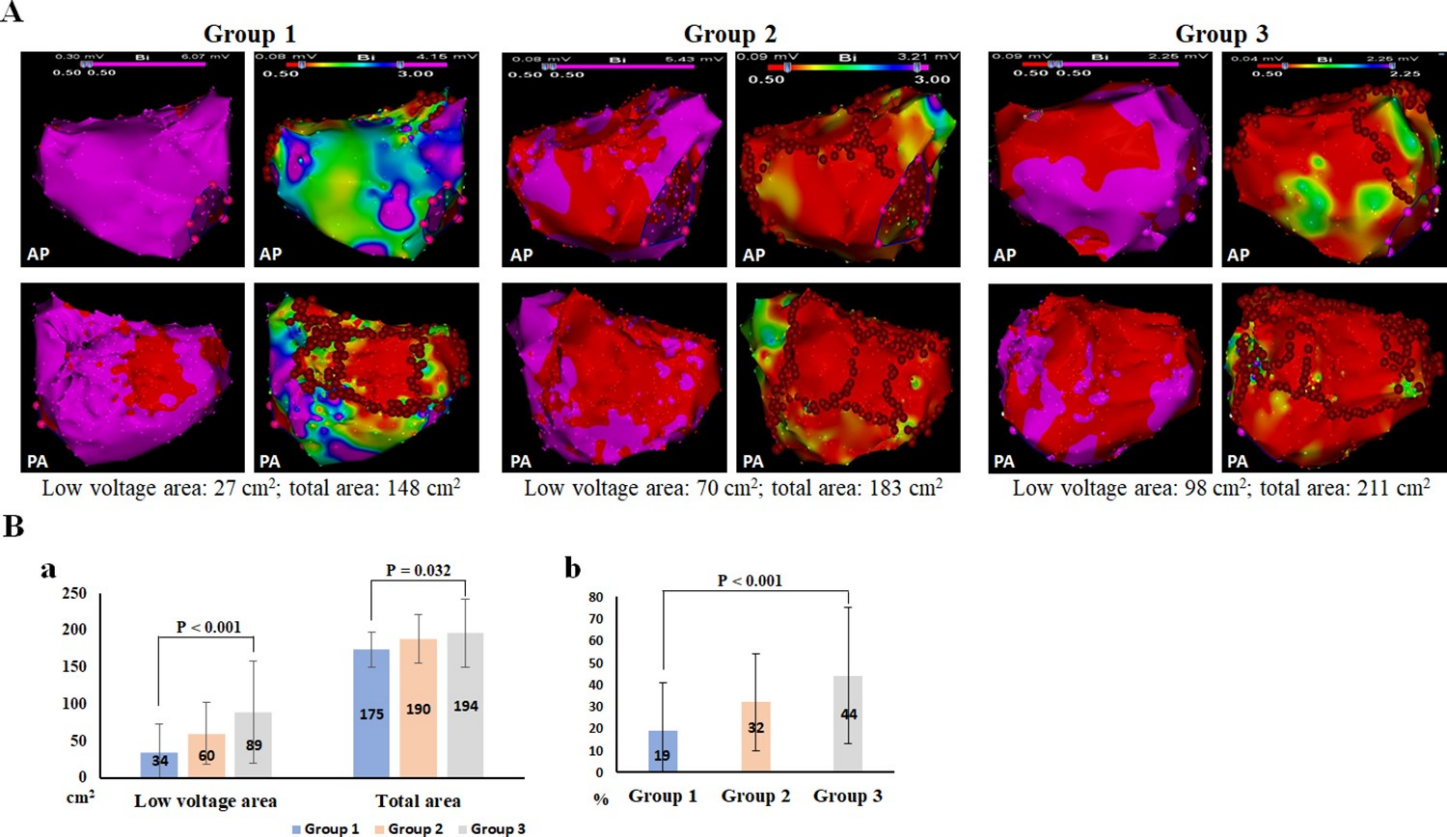
Examples of voltage maps and low-voltage area (LVA)-guided linear ablation in the three groups. A: Left subpanels show the distribution of LVA and right subpanels show linear ablation locations (red dots). As shown in right subpanels, the ablation lines were Compared with patients in Groups 2 and 3, patient in Group 1 showed a less extent of LVA and linear ablation was not performed at the mitral isthmus under LVA guidance. B: Summarized results of LVA (left), total left atrial area (right, subpanel a), and percentage of LVA relative to total left atrial area (subpanel b) in the three groups. AP, antero-posterior; PA: posteroanterior.

The extent of LVA was variable between patients ranging from 0.6% to 96.6% (mean 29.1%; median 21.8%) of the total LA surface area, and a high extent of LVA was associated with a poor prognosis. As shown in Fig 1A, the extent of LVA was lowest in Group 1 and highest in Group 3. The mean LVA was 34 ± 39, 60 ± 42, and 89 ± 70 mm^2^, and the mean ratio of LVA/LA total area was 19.4 ± 21.6, 31.9 ± 21.8, and 43.6 ± 30.7% in Groups 1, 2, and 3, respectively (*P* < 0.001 for both comparisons, Fig 1B).

Most patients underwent 2D echocardiographic examinations during sinus rhythm except in 4 patients with early recurrence of persistent AF on day 1 post ablation. In addition, the other 2 patients underwent 2D echocardiographic examinations during LAT at 3 months post ablation. Therefore, there were 141 of 145 (97.2%) and 139 of 145 (95.9%) patients undergoing echocardiography during sinus rhythm at day 1 and at 3 months post RFCA, respectively. The echocardiographic data are summarized in Table 2. Group 1 had the smallest LAD, LAV_max_, LAV_min_, and highest LAEF at day 1 post ablation. After 3 months, the differences became more significant among three groups. Unlike in Groups 1 and 2, LAEF in Group 3 did not improve 3 months after RFCA. Comparing the 1-day and 3-month post-RFCA data, the degree of reduction of LAD and LAV_min_, and the degree of increase in LAEF were significantly different among the three groups. This implies that LA reverse remodeling was related to the outcome status after AF ablation.

**Table 2:** Echocardiographic data of the study groups.

***1 day after ablation***					
Echocardiographic data	All patients (n = 145)	Group 1 (n = 63)	Group 2 (n = 53)	Group 3 (n = 29)	*p* value
LAD (mm)	46.7±6.1	44.9±5.2	47.7±6.2	48.9±6.6	.003
LAV_max_ (ml)	86.1±34.2	78.4±25.0	93.0±42.8	91.1±31.0	.048
LAV_min_ (ml)	54.7±30.0	44.9±20.2	60.6±36.9	65.2±27.8	.002
LAEF (%)	38.9±11.6	44.4±10.7	37.4±10.6	29.9±8.3	< .001
IVS (mm)	12.2±2.1	12.2±2.1	12.4±2.4	12.1±1.9	.814
LVEF (%)	63.1±9.4	64.6±5.7	61.1±12.3	63.6±9.4	.127
Peak A (cm/s)	42.2±22.2	40.4±16.1	44.5±20.0	42.1±36.1	.626
MR					.154
No or trivial	12 (8.3%)	8 (12.7%)	2 (3.8%)	2 (6.9%)	
Mild	82 (56.6%)	39 (61.9%)	29 (54.7%)	14 (48.3%)	
Mild to moderate	49 (33.8%)	16 (25.4%)	20 (37.7%)	13 (44.8%)	
≥ Moderate	2 (1.4%)	0 (0%)	2 (3.8%)	0 (0%)	
***3 months after ablation***					
Echocardiographic data	All patients	Group 1	Group 2	Group 3	*p* value
LAD (mm)	43.6±6.4	41.0±5.0	45.2±6.1	46.9±7.5	< .001
LAV_max_ (ml)	73.5±31.6	61.9±20.9	80.1±35.1	86.3±37.0	< .001
LAV_min_ (ml)	42.8±28.4	30.7±14.9	47.6±30.6	61.9±35.4	< .001
LAEF (%)	45.3±13.3	52.2±9.0	43.9±12.0	31.5±13.2	< .001
***Differences between post-ablation 1 day and 3 months***					
Echocardiographic data	All patients	Group 1	Group 2	Group 3	*p* value
ΔLAD (mm)	-3.1±3.4	-3.9±3.3	-2.7±3.6	-2.0±2.9	.030
ΔLAV_max_ (ml)	-13.0±19.9	-16.4±19.4	-12.9±20.1	-5.7±18.4	.057
ΔLAV_min_ (ml)	-12.1±15.6	-14.3±13.6	-13.6±16.7	-3.9±15.9	.011
ΔLAEF (%)	6.0±9.8	7.8±7.9	6.3±10.7	.83±10.9	.008

LAD: left atrial diameter; LAV_max_: maximal left atrial volume; LAV_min_: minimal left atrial volume; LAEF: left atrial emptying fraction; IVS: intraventricular septum; LVEF: left ventricular ejection fraction; MR: mitral regurgitation; ΔLAD, ΔLAV_max_, ΔLAV_min_, and ΔLAEF: differences of LAD, LAV_max_, LAV_min_, and LAEF between post-ablation 1 day and 3 months, respectively.

### Procedures of LA linear ablation

Tables 3 and 4 summarizes the number of patients undergoing LA linear ablation for various extents and locations. The mean number of ablation lines was highest in Group 3 (2.59 ± 1.53, 3.08 ± 1.31, and 3.31 ± 1.39 in Groups 1, 2, and 3, respectively, *P* = 0.048), and the ratio of patients undergoing different number of ablation lines were also significantly different among the three groups (Table 3, *P* = 0.038). That is, patients with a poorer ablation outcome underwent more lines of ablation. In 9 patients without undergoing LA linear ablation, 7 patients remained in sinus rhythm without AADs and 2 patients did not want to undergo repeat RFCA for AADs-refractory AF. In the remaining 136 patients who underwent LA linear ablation, the most common location was the roof, followed by anterior mitral isthmus, posterior mitral isthmus, posterior wall, and anteroseptum (Table 4). There were 125 patients underwent mitral isthmus ablation, including 69 (39%) patients undergoing both anterior and posterior approaches. Peri-mitral bidirectional block was achieved in 106 of 125 patients (85%), including 47 of 50 (94%) patients in Group 1, 45 of 48 (94%) patients in Group 2, and 14 of 27 (52%) patients in Group 3 (*P* < 0.001). It implies that peri-mitral bidirectional block remains an issue and should be accomplished for a better outcome of non-PAF ablation.

**Table 3:** Distribution of the percentage of patients undergoing different numbers of left atrial linear ablation among three groups.

No. of left atrial ablation line	Total (n = 145)	Group 1 (n = 63)	Group 2 (n = 53)	Group 3 (n = 29)
0	9 (6.2%)	7 (11.1%)	0 (0%)	2 (6.9%)
1	20 (13.8%)	7 (11.1%)	9 (17.0%)	4 (13.8%)
2	24 (16.6%)	14 (22.2%)	9 (17.0%)	1 (3.4%)
3	34 (23.4%)	16 (25.4%)	11 (20.8%)	7 (24.1%)
4	38 (26.2%)	11 (17.5%)	17 (32.1%)	10 (34.5%)
5	20 (13.8%)	8 (12.7%)	7 (13.2%)	5 (17.2%)

**Table 4:** Summary of patient numbers at different locations of left atrial linear ablation among three groups.

	Total (n = 145)	Group 1 (n = 63)	Group 2 (n = 53)	Group 3 (n = 29)	*P* value
Posterior MI	94 (64.8%)	40 (63.5%)	33 (62.3%)	21 (72.4%)	.627
Anterior MI	100 (69.0%)	35 (55.6%)	42 (79.2%)	23 (79.3%)	.009
Roof	114 (77.9%)	48 (76.2%)	44 (83.0%)	22 (75.9%)	.618
Posterior	67 (46.2%)	29 (46.0%)	24 (45.3%)	14 (48.3%)	.966
Anteroseptum	49 (33.8%)	16 (25.4%)	20 (37.7%)	13 (44.8%)	.140

MI, mitral isthmus.

### LVA-guided LA linear ablation rescues poor outcomes of RFCA in diseased left atria

**Fig 2:**
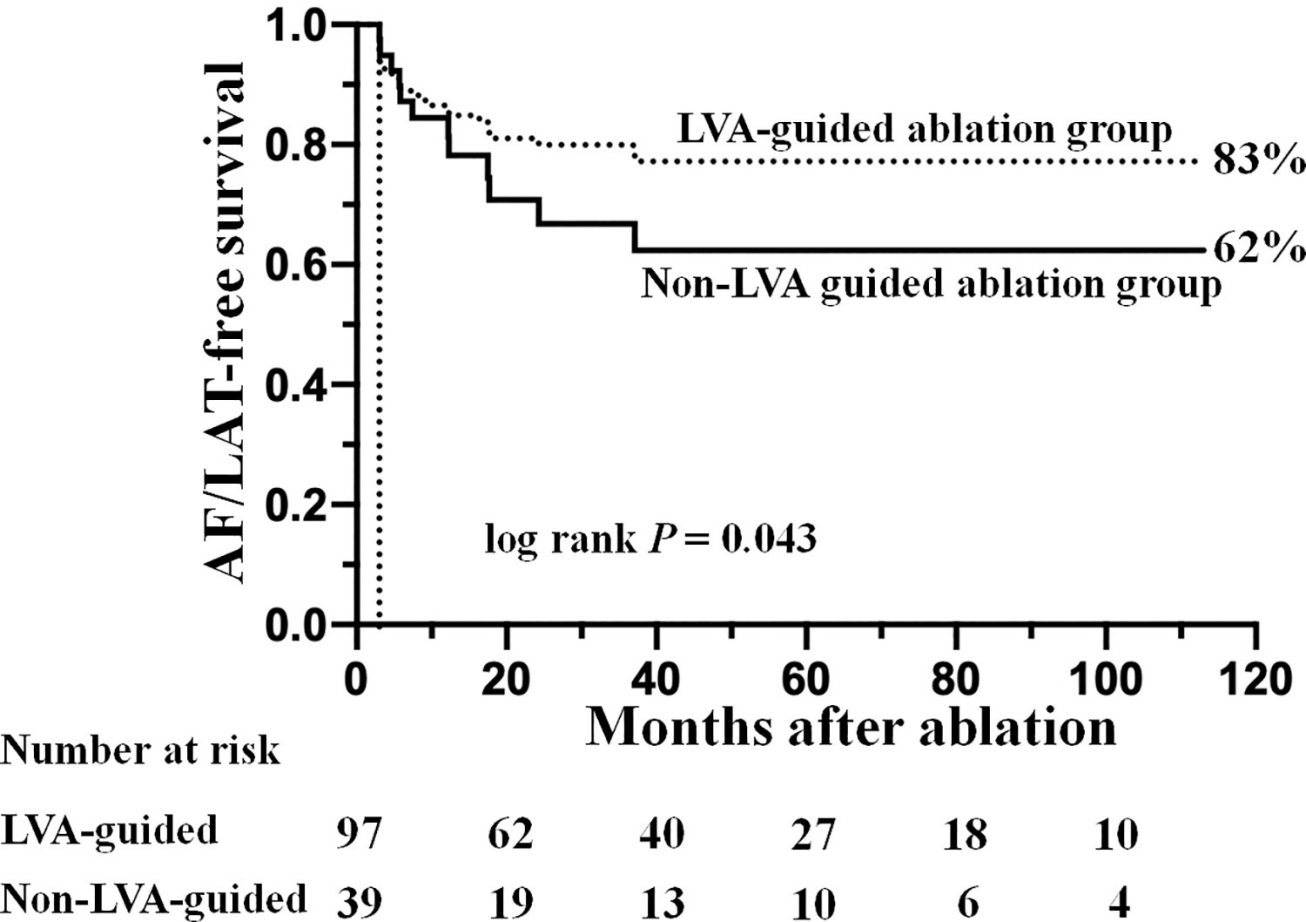
The Kaplan-Meier estimates of freedom from atrial fibrillation (AF)/left atrial tachycardia (LAT) in the low-voltage area (LVA)-guided (dashed line) and non-LVA-guided (solid-line) linear ablation groups.

In 136 patients undergoing LA linear ablation, LVA-guided linear ablation was performed in 97 (71%) patients; and the remaining patients included 16, 13, 6, and 4 patients undergoing 1, 2, 3, and 4 non-LVA-guided linear ablations, respectively. Baseline clinical characteristics of the two groups are summarized in Table 5. The LVA-guided ablation group patients were older, more female, and had smaller body-mass index than those in the non-LVA-guided ablation group. The non-LVA-guided linear ablation lesions were located at anterior mitral isthmus (n = 27, 27%), posterior mitral isthmus (n = 30, 32%), roof (n = 6, 5%), posterior wall (n = 3, 4%), and anteroseptum (n = 10, 20%). Acute success in creating linear block at the roof and posterior wall could be achieved by LVA- or non-LVA-guided ablation in all patients. But the main challenge was to create linear block at the mitral isthmus. For posterior mitral isthmus, LVA- and non-LVA-guided ablation created linear block in 41 of 64 (64%) and 8 of 30 (27%) patients, respectively (*P* < 0.001); for anterior mitral isthmus, LVA- and non-LVA- guided ablation created linear block in 63 of 73 (86%) and 17 of 27 (63%) patients, respectively (*P* = 0.01). Table 6 summarizes the characteristics of the left atrium in patients undergoing LVA- and non-LVA-guided linear ablation. The LVA-guided group had a significantly higher extent of LVA (*P* < 0.001), and larger LAD (*P* < 0.001), LAV_max_ (*P* = 0.009), and LAV_min_ (*P* = 0.007) than the non-LVA-guided group. However, the long-term AF/LAT-free survival rates on or off AADs were 83% vs. 62% for LVA- and non-LVA-guided groups, respectively (*P* = 0.043, Fig 2). It implies that LVA-guided LA linear ablation rescued a poor outcome of RFCA in non-PAF patients with a diseased left atrium.

**Table 5:** Baseline characteristics, clinical data, and ablation results of the LVA-and non-LVA guided linear ablation groups.

	LVA	Non-LVA	*p* value
Patient number	97 (71%)	39 (29%)	
Age (years)	60±13	53±11	.006
Gender (male, %)	62 (64%)	35 (90%)	.003
BMI (kg/m^2^)	26.0±3.3	27.7±4.1	.015
AFD (years)	3.85±4.46	4.51±3.82	.325
CHA_2_DS_2_-VASc	1.86±1.42	1.38±1.29	.075
Hypertension (%)	52 (54%)	20 (51%)	.851
Diabetes mellitus (%)	16 (17%)	9 (23%)	.463
Dyslipidemia (%)	40 (41%)	14 (36%)	.699
CAD (%)	4 (4%)	0 (0%)	.578
Stroke (%)	11 (11%)	6 (15%)	.570
ESRD (%)	2 (2%)	0 (0%)	1.000
RHD (%)	6 (0%)	1 (3%)	.673
SSS (%)	17 (18%)	6 (15%)	1.000
COPD (%)	3 (3%)	1 (3%)	1.000
Smoking (%)	5 (5%)	6 (15%)	.077
RFCA times	1.42±0.75	1.62±0.71	.171
AF conversion	40 (41%)	18 (46%)	.702

AFD: atrial fibrillation duration; BMI: body-mass index; CAD: coronary artery disease; COPD: chronic obstructive pulmonary disease; ESRD: end-stage renal disease; LVA: low-voltage area; RFCA: radiofrequency catheter ablation RHD: rheumatic heart disease; SSS: sick sinus syndrome.

**Table 6:** Summarized results of left atrial characteristics in patients undergoing LVA- and non-LVA-guided left atrial linear ablation.

	Total patients	LVA-guided	non-LVA-guided	*P* values
Patient number	136	97 (71%)	39 (29%)	
LVA (cm^2^)	57.5±55.3	71.6±57.7	22.5±26.1	< .001
LAA (cm^2^)	185.0±35.9	188.7±38.2	175.9±28.1	0.060
LVA%	29.1±24.7	35.5±24.6	13.6±16.8	< .001
LAD (mm)	47.0±5.9	47.7±6.2	45.3±4.9	0.035
LAV_max_ (ml)	87.1±34.3	91.9±36.7	75.1±24.0	0.009
LAV_min_ (ml)	55.3±29.9	59.7±32.5	44.5±18.6	0.007
LAEF (%)	38.6±11.4	37.6±11.2	41.3±11.5	0.082
LAD_3m (mm)	43.8±6.2	44.3±6.3	42.7±6.0	0.176
LAV_max__3m (ml)	74.4±31.1	78.6±33.3	64.0±21.8	0.012
LAV_min__3m (ml)	43.2±27.8	46.6±29.6	34.6±19.8	0.022
LAEF_3m (%)	44.9±13.0	43.7±13.1	47.9±12.4	0.083
ΔLAD (mm)	-3.2±3.4	-3.4±3.7	-2.6±2.5	0.246
ΔLAV_max_ (ml)	-12.6±19.6	-13.3±19.3	-11.1±20.7	0.562
ΔLAV_min_ (ml)	-12.1±15.8	-13.1±16.5	-9.6±13.7	0.249
ΔLAEF (%)	6.3±10.0	6.1±10.1	6.6±9.8	0.789

LA, left atrium; LAA, LA total area; LVA, low-voltage area; LVA% = percentage of LVA relative to LAA; LAD: left atrial diameter; LAV_max_: maximal left atrial volume; LAV_min_: minimal left atrial volume; LAEF: left atrial emptying fraction; LAD_3m, LAV_max__3m, LAV_min__3m, LAEF_3m: data at 3 months after ablation; ΔLAD, ΔLAV_max_, ΔLAV_min_, and ΔLAEF: differences of LAD, LAV_max_, LAV_min_, and LAEF between post-ablation 1 day and 3 months, respectively.

### Effects of the extent of linear ablation on LA reverse remodeling

As shown in Table 7, patients with a larger LAV_max_ (*P* = 0.012), LAV_min_ (*P* = 0.003), and a lower LAEF (*P* < 0.001) at 1 day post ablation underwent more LA linear ablations. We further examined the impacts of the extent of linear ablation on LA reverse remodeling. LAD, LAV_max_, and LAV_min_ were reduced and LAEF was improved 3 months after AF ablation in all subgroups, and the differences in LAD (*P* = 0.780), LAV_max_ (*P* = 0.064), LAV_min_ (*P* = 0.081), and LAEF (*P* = 0.742) between the post-ablation 1 day and 3 months were all insignificant among the subgroups undergoing various extents of linear ablation. That is, the adverse effects of extensive linear ablation were outweighed by LA reverse remodeling even if multiple linear ablations were performed in more diseased left atria.

**Table 7:** Echocardiographic data in patients underwent different number of left atrial linear ablation lines.

No. of linear ablation line	0	1	2	3	4	5	*p* value
	(n = 9)	(n = 20)	(n = 24)	(n = 34)	(n = 38)	(n = 20)	
***1 day after ablation***							
LAD (mm)	42.8±7.2	46.4±6.7	45.1±5.0	47.0±6.2	47.7±6.2	48.5±5.1	.138
LAV_max_ (ml)	74.2±31.2	69.5±20.4	78.7±23.3	85.1±29.8	95.3±39.3	102.4±43.8	.012
LAV_min_ (ml)	45.0±29.7	39.5±17.0	43.5±19.1	55.6±24.2	64.4±35.4	67.9±37.4	.003
LAEF (%)	43.4±14.5	44.9±11.1	46.0±11.5	36.5±9.7	34.4±9.4	35.2±11.7	<0.001
***3 months after ablation***							
LAD (mm)	40.8±7.5	42.4±6.2	42.3±5.2	43.6±5.8	44.8±7.1	45.6±6.4	.282
LAV_max_ (ml)	60.7±27.4	67.0±21.9	65.2±20.8	68.9±20.2	84.3±40.5	83.7±38.0	.038
LAV_min_ (ml)	33.9±30.4	35.2±18.2	32.8±15.9	39.3±17.0	52.3±35.0	52.9±37.3	.013
LAEF (%)	50.1±16.1	49.8±13.5	50.5±10.7	44.3±12.5	41.1±12.4	41.6±14.3	.016
***Differences between post-ablation 1 day and 3 months***							
ΔLAD (mm)	-2.0±3.0	-4.0±4.5	-2.8±3.3	-3.4±3.2	-2.9±2.9	-2.9±3.7	.780
ΔLAV_max_ (ml)	-13.4±24.0	-2.5±14.2	-13.5±14.1	-16.3±22.3	-11.0±18.8	-18.7±23.7	.064
ΔLAV_min_ (ml)	-11.1±13.6	-4.3±10.7	-10.7±11.9	-16.3±17.3	-11.9±16.8	-15.0±17.5	.081
ΔLAEF (%)	6.7±9.0	4.9±11.1	4.5±7.8	7.8±9.0	6.6±11.9	6.4±9.1	.742

LAD: left atrial diameter; LAV_max_: maximal left atrial volume; LAV_min_: minimal left atrial volume; LAEF: left atrial emptying fraction; IVS: intraventricular septum; LVEF: left ventricular ejection fraction; MR: mitral regurgitation. ΔLAD, ΔLAV_max_, ΔLAV_min_, and ΔLAEF: differences of LAD, LAV_max_, LAV_min_, and LAEF between post-ablation 1 day and 3 months, respectively.

## Discussion

In this study, additional LA linear ablation for non-PAF was associated with a AADs-refractory AF/LAT recurrence rate of 20% after a mean follow-up duration of 48 ± 33 months. Low LA emptying fraction, large LA size and high extent of LVA were associated with AF recurrence. LVA-guided linear ablation was performed in 71% patients who had a higher extent of LVA and more dilated left atrium than patients undergoing non-LVA-guided linear ablation, but the long-term AF/LAT-free survival rate was higher in the LVA-guided group. The rate of acute success in creating linear block at the mitral isthmus was significantly higher via LVA-guided than non-LVA-guided linear ablation. LA reverse remodeling after resuming sinus rhythm was noted even in patients with a diseased left atrium undergoing extensive LA linear ablation. This strategy has the advantages of avoiding ablation on healthy atrial tissues and targeting the diseased arrhythmogenic atrial tissues to rescue a poor RFCA outcome in non-PAF patients with a diseased left atrium.

### LVA-guided LA linear ablation for non-PAF

CPVI is effective for AF suppression if the focal mechanism with fibrillatory conduction is the predominant mechanism for AF maintenance [19]. Because persistence of AF results in progressive LA dilatation, fibrosis and self-perpetuation of AF [20, 21], additional linear ablation for LA mass reduction has been considered to prevent AF/LAT recurrence in non-PAF [22]. In a prospective randomized study conducted by Willems and colleagues, 69% of patients with non-PAF undergoing additional linear ablations at LA roof and posterior mitral isthmus remained in sinus rhythm, compared with only 20% of patients receiving PV isolation only [1]. In that study, the posterior mitral line was attempted to connect both posterior mitral annulus and left inferior pulmonary vein using the shortest distance. Because of the thickness of atrial myocardium (up to 8 mm) [23], the high amount of radiofrequency applications and difficulty in achieving durable complete conduction block across the posterior mitral isthmus remained issues. Pak et al. reported that the presence of LVA on the anterior wall resulted in a better clinical outcome for peri-mitral bidirectional block via LA anterior wall approach than ablation on the thick lateral ridge [24]. Consistently, our data showed that LVA- and non-LVA-guided ablation created linear block at the posterior mitral isthmus in 41 of 64 (64%) and 8 of 30 (27%) patients, respectively; and at the anterior mitral isthmus in 63 of 73 (86%) and 17 of 27 (63%) patients, respectively. LVA-guided approach did help achieving a significantly higher rate of acute success in creating linear block at the mitral isthmus than non-LVA-guided approach. A recent meta-analysis by Blandino et al. showed that LVA-guided ablation was more effective than CPVI to prevent AF/LAT recurrence in the treatment of non-PAF patients with diseased atrial tissues [25]. Our data showed that LVA-guided linear ablation was performed in more diseased left atria but with a higher long-term AF/LAT-free survival rate than non-LVA-guided linear ablation. It implies that linear lesions created with consideration of the underlying atrial substrate would be superior to empirically defined linear ablation for LA mass reduction in non-PAF patients.

### Strategies of LVA-targeted ablation for non-PAF patients

There have been studies evaluating LVA ablation or creating different types of anatomic lines to isolate LVA [5, 10–12, 26, 27], but consensus has not been reached on the optimal LVA-targeted strategy to treat non-PAF yet. Jadidi et al. reported that ablation at LVA< 0.5 mV in AF with fractionated activity or discrete rapid local activity is more effective with reduced radiofrequency delivery than conventional CPVI [5]. Efremidis et al. reported that ablating LVA < 0.5 mV in AF with specific electrogram characteristics showing rotational activity or activation gradient covering greater than 70% of AF cycle length after CPVI is associated with good outcomes, particularly when AF termination is achieved [12]. Regional LVA ablation and homogenization has been reported to improve the long-term success rate in non-PAF patients [11], but strategic linear ablation is frequently needed to connect the ablated LVA to anatomical obstacles because LVA homogenization alone cannot prevent rotor anchoring [27]. Alternatively, our study focused on linear ablation across LVA to eliminate arrhythmogenic atrial tissues as well as to create lines of block to prevent reentry formation. We did not intend to create an ablation line to divide the low potential region because the ablation lines could be across or at the border of LVA. Our data showed that LVA-guided linear ablation had a better long-term AF/LAT-free survival rate than non-LVA-guided linear ablation even if the LVA-guided group had higher extent LVA and more diseased left atria. The combination of targeting the arrhythmogenic LVA and LA mass reduction would be an alternative individually tailored approach for non-PAF ablation.

We have used voltage mapping during ongoing AF [5]. It had the advantage that the spatial distribution of mean voltage during AF better correlates with delay enhancement magnetic resonance imaging-detected atrial fibrosis than with sinus rhythm, and arrhythmogenic vulnerabilities associated with fibrosis that are dormant during sinus rhythm may become manifest during the functional circumstances encompassing AF [28]. However, this cutoff value of 0.5 mV for defining LVA during AF corresponds to LVA < 1.0 mV when mapping is performed during sinus rhythm. That is, LVA defined during AF is larger than that defined during sinus rhythm with a same cutoff value. Rodríguez-Mañero et al. reported that a cutoff of 0.31 mV for AF predicts a sinus voltage of 0.5 mV [29]. It is still unknown the exact voltage threshold to be considered pathological in AF or sinus rhythm. To preserve LA tissue from ablation, bipolar voltage cutoffs should be adjusted depending on the rhythm, especially when regional LVA ablation and homogenization is the strategy for LA substrate modification. Recent findings by Jadidi et al. indicate that prolonged delayed potentials in sinus rhythm correspond to continuous, rapid activities in AF that display low voltages [30]. A sinus rhythm-based mapping approach for identification of low-voltage and slow conduction substrate might be an alternative approach that allows to preserve more LA tissue than mapping and ablation during AF.

### LA reverse remodeling with LVA-guided linear ablation

In this study, significant LA reverse remodeling was evidenced by improved LAEF and reduced LA size in 3-month follow-up cardiac echo in patients with no recurrence of AF/LAT even if extensive linear ablation was performed in diseased left atria. This result is in line with previous reports that sinus rhythm maintenance brings histological reverse remodeling and functional LA recovery that overwhelm the harmful effects of iatrogenic scaring by extensive linear ablation.[31, 32] Theoretically targeting LVA can avoid injury to other healthy atrial myocardium and preserve LA function and thus is supported to induce a greater degree of LA reverse remodeling. However, our data did not show significant differences in the reduction of LA size or the increase of LAEF between the LVA-guided and non-LVA-guided groups. A possible explanation is that preexisting advanced LA myopathy revealed by a higher extent of LVA and more dilated left atrium in the LVA-guided group interfered with LA reverse remodeling in this study population.

### Limitations

This was a retrospective study with small size that could bias patient characteristics and limit the statistical accuracy of our results. Multicenter randomized controlled trials in sufficient numbers of patients are needed to clarify the role of LVA-guided linear ablation in non-PAF patients. To confirm the sites as non-pulmonary vein triggers of AF, it is necessary to document that AF is triggered by these foci and ablation on these foci results in AF termination. However, we did not cardiovert AF into sinus rhythm to find the triggers before ablation, and linear ablation rather than single point ablation was performed in this study. Therefore, we did not exactly know how many non-pulmonary vein triggers were ablated. We routinely performed cardiac CT but not delayed enhancement cardiac magnetic resonance imaging in all patients before ablation. Therefore, we cannot comment for sure that the low-potential region includes scar sites in this study. Our study identified LVA in AF rather than in sinus rhythm. The presented data should be interpreted with caution when LVA is identified in sinus rhythm. AF recurrence was quantified on the basis of patient symptoms, therefore, freedom from AF and LAT is likely overestimated as systematic extended monitoring was not performed in all patients to reveal asymptotic paroxysmal tachycardias.

## Conclusion

Additional LVA-guided linear ablation through targeting the arrhythmogenic LVA and reducing LA mass is better than non-LVA guided linear ablation to prevent AF recurrence in non-PAF patients. LA reverse remodeling occurred in patients with sinus rhythm maintenance after RFCA regardless of the extent of linear ablation.

## Supporting information

S1 File Data file of patients underwent LVA-guided linear ablation and outcome. (SAV)

## References

[1] Willems S, Klemm H, Rostock T, Brandstrup B, Ventura R, Steven D, et al. Substrate modification combined with pulmonary vein isolation improves outcome of catheter ablation in patients with persistent atrial fibrillation: a prospective randomized comparison. Eur Heart J. 2006;27(23):2871–8. doi: 10.1093/eurheartj/ehl093 16782716

[2] Fassini G, Riva S, Chiodelli R, Trevisi N, Berti M, Carbucicchio C, et al. Left mitral isthmus ablation associated with PV isolation: long‐term results of a prospective randomized study. J Cardiovasc Electrophysiol. 2005;16(11):1150–6. doi: 10.1111/j.1540-8167.2005.50192.x 16302895

[3] Brooks AG, Stiles MK, Laborderie J, Lau DH, Kuklik P, Shipp NJ, et al. Outcomes of long-standing persistent atrial fibrillation ablation: a systematic review. Heart Rhythm. 2010;7(6):835–46. doi: 10.1016/j.hrthm.2010.01.017 20206320

[4] Verma A, Jiang C-y, Betts TR, Chen J, Deisenhofer I, Mantovan R, et al. Approaches to catheter ablation for persistent atrial fibrillation. N Engl J Med. 2015;372(19):1812–22. doi: 10.1056/NEJMoa1408288 25946280

[5] Jadidi AS, Lehrmann H, Keyl C, Sorrel J, Markstein V, Minners J, et al. Ablation of persistent atrial fibrillation targeting low-voltage areas with selective activation characteristics. Circ Arrhythm Electrophysiol. 2016;9(3):e002962. doi: 10.1161/CIRCEP.115.002962 26966286

[6] Verma A, Wazni OM, Marrouche NF, Martin DO, Kilicaslan F, Minor S, et al. Pre-existent left atrial scarring in patients undergoing pulmonary vein antrum isolation: an independent predictor of procedural failure. J Am Coll Cardiol. 2005;45(2):285–92. doi: 10.1016/j.jacc.2004.10.035 15653029

[7] Vlachos K, Efremidis M, Letsas KP, Bazoukis G, Martin R, Kalafateli M, et al. Low‐voltage areas detected by high‐density electroanatomical mapping predict recurrence after ablation for paroxysmal atrial fibrillation. J Cardiovasc Electrophysiol. 2017;28(12):1393–402. doi: 10.1111/jce.13321 28884923

[8] Masuda M, Asai M, Iida O, Okamoto S, Ishihara T, Nanto K, et al. Additional Low‐Voltage‐Area Ablation in Patients With Paroxysmal Atrial Fibrillation: Results of the Randomized Controlled VOLCANO Trial. J Am Heart Assoc. 2020;9(13):e015927. doi: 10.1161/JAHA.120.015927 32578466 PMC7670527

[9] Rolf S, Kircher S, Arya A, Eitel C, Sommer P, Richter S, et al. Tailored atrial substrate modification based on low-voltage areas in catheter ablation of atrial fibrillation. Circ Arrhythm Electrophysiol. 2014;7(5):825–33. doi: 10.1161/CIRCEP.113.001251 25151631

[10] Kottkamp H, Berg J, Bender R, Rieger A, Schreiber D. Box isolation of fibrotic areas (BIFA): A patient‐tailored substrate modification approach for ablation of atrial fibrillation. J Cardiovasc Electrophysiol. 2016;27(1):22–30. doi: 10.1111/jce.12870 26511713

[11] Yamaguchi T, Tsuchiya T, Nakahara S, Fukui A, Nagamoto Y, Murotani K, et al. Efficacy of left atrial voltage‐based catheter ablation of persistent atrial fibrillation. J Cardiovasc Electrophysiol. 2016;27(9):1055–63. doi: 10.1111/jce.13019 27235000

[12] Efremidis M, Vlachos K, Letsas KP, Bazoukis G, Martin R, Frontera A, et al. Targeted ablation of specific electrogram patterns in low‐voltage areas after pulmonary vein antral isolation in persistent atrial fibrillation: Termination to an organized rhythm reduces atrial fibrillation recurrence. J Cardiovasc Electrophysiol. 2019;30(1):47–57. doi: 10.1111/jce.13763 30288830

[13] Jeevanantham V, Ntim W, Navaneethan SD, Shah S, Johnson AC, Hall B, et al. Meta-analysis of the effect of radiofrequency catheter ablation on left atrial size, volumes and function in patients with atrial fibrillation. Am J Cardiol. 2010;105(9):1317–26. doi: 10.1016/j.amjcard.2009.12.046 20403486

[14] Packer M. Effect of catheter ablation on pre-existing abnormalities of left atrial systolic, diastolic, and neurohormonal functions in patients with chronic heart failure and atrial fibrillation. Eur Heart J. 2019;40(23):1873–9. doi: 10.1093/eurheartj/ehz284 31081029 PMC6568203

[15] Calkins H, Hindricks G, Cappato R, Kim Y-H, Saad EB, Aguinaga L, et al. 2017 HRS/EHRA/ECAS/APHRS/SOLAECE expert consensus statement on catheter and surgical ablation of atrial fibrillation. Europace. 2018;20(1):e1–e160. doi: 10.1093/europace/eux274 29016840 PMC5834122

[16] Chou C-C, Lee H-L, Chang P-C, Wo H-T, Wen M-S, Yeh S-J, et al. Left atrial emptying fraction predicts recurrence of atrial fibrillation after radiofrequency catheter ablation. PLoS One. 2018;13(1):e0191196. doi: 10.1371/journal.pone.0191196 29364912 PMC5783382

[17] Jaïs P, Hocini M, Hsu L-F, Sanders P, Scavee C, Weerasooriya R, et al. Technique and results of linear ablation at the mitral isthmus. Circulation. 2004;110(19):2996–3002. doi: 10.1161/01.CIR.0000146917.75041.58 15520313

[18] Hwang Y-T, Lee H-L, Lu C-H, Chang P-C, Wo H-T, Liu H-T, et al. A Novel Approach for Predicting Atrial Fibrillation Recurrence After Ablation Using Deep Convolutional Neural Networks by Assessing Left Atrial Curved M-Mode Speckle-Tracking Images. Front Cardiovasc Med. 2021; 7:605642. doi: 10.3389/fcvm.2020.605642 33553257 PMC7862331

[19] Mandapati R, Skanes A, Chen J, Berenfeld O, Jalife J. Stable microreentrant sources as a mechanism of atrial fibrillation in the isolated sheep heart. Circulation. 2000;101(2):194–9. doi: 10.1161/01.cir.101.2.194 10637208

[20] den Uijl DW, Cabanelas N, Benito EM, Figueras R, Alarcon F, Borras R, et al. Impact of left atrial volume, sphericity, and fibrosis on the outcome of catheter ablation for atrial fibrillation. J Cardiovasc Electrophysiol. 2018;29(5):740–6. doi: 10.1111/jce.13482 29528532

[21] Habibi M, Lima JA, Khurram IM, Zimmerman SL, Zipunnikov V, Fukumoto K, et al. Association of left atrial function and left atrial enhancement in patients with atrial fibrillation: cardiac magnetic resonance study. Circ Cardiovasc Imaging. 2015;8(2):e002769. doi: 10.1161/CIRCIMAGING.114.002769 25652181 PMC4319560

[22] Knecht S, Hocini M, Wright M, Lellouche N, O’Neill MD, Matsuo S, et al. Left atrial linear lesions are required for successful treatment of persistent atrial fibrillation. Eur Heart J. 2008;29(19):2359–66. doi: 10.1093/eurheartj/ehn302 18614522

[23] Wittkampf FH, van Oosterhout MF, Loh P, Derksen R, Vonken E-j, Slootweg PJ, et al. Where to draw the mitral isthmus line in catheter ablation of atrial fibrillation: histological analysis. Eur Heart J. 2005;26(7):689–95. doi: 10.1093/eurheartj/ehi095 15637084

[24] Pak H-N, Oh YS, Lim HE, Kim Y-H, Hwang C. Comparison of voltage map-guided left atrial anterior wall ablation versus left lateral mitral isthmus ablation in patients with persistent atrial fibrillation. Heart Rhythm. 2011;8(2):199–206. doi: 10.1016/j.hrthm.2010.10.015 20950713

[25] Blandino A, Bianchi F, Grossi S, BIONDI‐ZOCCAI G, Conte MR, Gaido L, et al. Left atrial substrate modification targeting low‐voltage areas for catheter ablation of atrial fibrillation: a systematic review and meta‐analysis. Pacing Clin Electrophysiol. 2017;40(2):199–212. doi: 10.1111/pace.13015 28054377

[26] Cutler MJ, Johnson J, Abozguia K, Rowan S, Lewis W, Costantini O, et al. Impact of voltage mapping to guide whether to perform ablation of the posterior wall in patients with persistent atrial fibrillation. J Cardiovasc Electrophysiol. 2016;27(1):13–21. doi: 10.1111/jce.12830 26515166

[27] Kircher S, Arya A, Altmann D, Rolf S, Bollmann A, Sommer P, et al. Individually tailored vs. standardized substrate modification during radiofrequency catheter ablation for atrial fibrillation: a randomized study. Europace. 2018;20(11):1766–75. doi: 10.1093/europace/eux310 29177475

[28] Qureshi NA, Kim SJ, Cantwell CD, Afonso VX, Bai W, Ali RL, et al. Voltage during atrial fibrillation is superior to voltage during sinus rhythm in localizing areas of delayed enhancement on magnetic resonance imaging: an assessment of the posterior left atrium in patients with persistent atrial fibrillation. Heart rhythm. 2019;16(9):1357–67. doi: 10.1016/j.hrthm.2019.05.032 31170484 PMC6722483

[29] Rodríguez-Mañero M, Valderrábano M, Baluja A, Kreidieh O, Martínez-Sande JL, García-Seara J, et al. Validating left atrial low voltage areas during atrial fibrillation and atrial flutter using multielectrode automated electroanatomic mapping. JACC Clin Electrophysiol. 2018;4(12):1541–52. doi: 10.1016/j.jacep.2018.08.015 30573117

[30] Jadidi A, Nothstein M, Chen J, Lehrmann H, Dössel O, Allgeier J, et al. Specific electrogram characteristics identify the extra-pulmonary vein arrhythmogenic sources of persistent atrial fibrillation–characterization of the arrhythmogenic electrogram patterns during atrial fibrillation and sinus rhythm. Sci Rep. 2020;10(1):1–12. doi: 10.1038/s41598-019-56847-4 32499483 PMC7272441

[31] Machino-Ohtsuka T, Seo Y, Ishizu T, Yanaka S, Nakajima H, Atsumi A, et al. Significant improvement of left atrial and left atrial appendage function after catheter ablation for persistent atrial fibrillation. Circ J. 2013;77(7):1695–704. doi: 10.1253/circj.cj-12-1518 23535197

[32] Takahashi Y, O’Neill MD, Hocini M, Reant P, Jonsson A, Jaïs P, et al. Effects of stepwise ablation of chronic atrial fibrillation on atrial electrical and mechanical properties. J Am Coll Cardiol. 2007;49(12):1306–14. doi: 10.1016/j.jacc.2006.11.033 17394963

